# Prospective association between an obesogenic dietary pattern in early adolescence and metabolomics derived and traditional cardiometabolic risk scores in adolescents and young adults from the ALSPAC cohort

**DOI:** 10.1186/s12986-023-00754-z

**Published:** 2023-09-15

**Authors:** Eduard Martínez Solsona, Laura Johnson, Kate Northstone, Genevieve Buckland

**Affiliations:** 1 Centre for Exercise, Nutrition and Health Sciences, School for Policy Studies, University of Bristol, 8 Priory Road, BS8 1TZ Bristol, UK; 2 Population Health Sciences, Bristol Medical School, University of Bristol, Bristol, UK; 3 Centre for Health, NatCen Social Research, London, UK; 4 Centre for Academic Child Health, Bristol Medical School, University of Bristol, Bristol, UK

**Keywords:** Cardiometabolic risk, Metabolomics, Dietary pattern, Adolescence, ALSPAC

## Abstract

**Background:**

Dietary intake during early life may be a modifying factor for cardiometabolic risk (CMR). Metabolomic profiling may enable more precise identification of CMR in adolescence than traditional CMR scores. We aim to assess and compare the prospective associations between an obesogenic dietary pattern (DP) score at age 13 years with a novel vs. traditional CMR score in adolescence and young adulthood in the Avon Longitudinal Study of Parents and Children (ALSPAC).

**Methods:**

Study participants were ALSPAC children with diet diary data at age 13. The obesogenic DP z-score, characterized by high energy-density, high % of energy from total fat and free sugars, and low fibre density, was previously derived using reduced rank regression. CMR scores were calculated by combining novel metabolites or traditional risk factors (fat mass index, insulin resistance, mean arterial blood pressure, triacylglycerol, HDL and LDL cholesterol) at age 15 (n = 1808), 17 (n = 1629), and 24 years (n = 1760). Multivariable linear regression models estimated associations of DP z-score with log-transformed CMR z-scores.

**Results:**

Compared to the lowest tertile, the highest DP z-score tertile at age 13 was associated with an increase in the metabolomics CMR z-score at age 15 (β = 0.20, 95% CI 0.09, 0.32, p trend < 0.001) and at age 17 (β = 0.22, 95% CI 0.10, 0.34, p trend < 0.001), and with the traditional CMR z-score at age 15 (β = 0.15, 95% CI 0.05, 0.24, p trend 0.020). There was no evidence of an association at age 17 for the traditional CMR z-score (β = 0.07, 95% CI -0.03, 0.16, p trend 0.137) or for both scores at age 24.

**Conclusions:**

An obesogenic DP was associated with greater CMR in adolescents. Stronger associations were observed with a novel metabolite CMR score compared to traditional risk factors. There may be benefits from modifying diet during adolescence for CMR health, which should be prioritized for further research in trials.

**Supplementary Information:**

The online version contains supplementary material available at 10.1186/s12986-023-00754-z.

## Introduction

Cardiovascular diseases (CVD) remain the leading cause of mortality and morbidity in the world, and with increasing deaths and disability-adjusted life years [1]. CVD develops gradually throughout the lifespan and precursors of CVD, such as early signs of atherosclerosis and elevated inflammatory conditions, have already been found among adolescents [2] and children [2–4]. Key modifiable CVD risk factors include an accumulated exposure to tobacco and poor diets, and a high body mass index (BMI) and sedentary lifestyle [5]. These can lead to a combination of metabolic dysfunctions such as insulin resistance, impaired glucose tolerance, dyslipidemia and hypertension [6].

Poor diet quality during childhood/adolescence is associated with metabolic risk factors of CVD in adolescence/young adults [7–10]. Assessing dietary patterns (DP), as opposed to isolated nutrients or foods, can better estimate diet-disease associations as it captures the way foods and nutrients are eaten in combination and possible interactions between them [11]. Cardioprotective DPs like those based on the Mediterranean Diet [12], Diet Approaches to Stop Hypertension, [13], and Alternative Healthy Eating Index [14] are recommended for CVD prevention [15–17]. However, these DPs are typically defined based on dietary guidelines and represent an optimal way of eating that may not be commonly adhered to in general populations. Furthermore, culturally specific scores like the Mediterranean diet have low adherence in the UK [18]. Data driven DP analysis allows local food intake patterns to be defined [19]. Reduced rank regression (RRR) specifies the nutrient mechanisms linking food intake to diseases typically identifying DPs with stronger associations with disease risk because the score captures disease-specific variation in diet rather than all variation [20]. An obesogenic DP that is low in fibre, energy dense, high in fat and free sugars identified early in life was associated with adiposity [21] and with conventional CMR factors (glucose, waist circumference, BMI, insulin, HDL-c, LDL-c, triglycerides) in adolescents [10]. Similar obesogenic DPs have been observed in adults in the UK National Diet and Nutrition Survey [22] and have been associated with the incidence of type 2 diabetes [23] and cardiovascular disease [24] in the UK Biobank. However, it is unknown how associations of an obesogenic DP with CMR evolve from childhood to adulthood [10, 25].

Accurately evaluating conventional CMR factors in children/young adults can be challenging due to age-related differences in hormones, metabolism, comorbidities and pathogenic pathways [26, 27]. Thus, new potential biomarkers of early CMR are needed [27, 28]. Metabolomics provides measurement of all metabolites, which potentially allows more precise identification of dietary associations with metabolic traits and later diseases [29, 30]. Deelen et al., [31], using a metabolomics approach, identified a 14-metabolite score mainly from lipid metabolism, lipoprotein and fatty acids, that had stronger associations with mortality than a conventional risk factors score in 44.,168 adults from 9 cohorts. All these 14 metabolites were individually associated with cardiovascular-related mortality or CMR in the same study or in previous research [31–41]. Therefore, evidence from adults suggests that metabolomics is a promising method to assess associations with CMR. Studies using metabolite-based CMR scores in adolescents are scarce although they could elucidate mechanisms linking dietary habits early in life with developing CVD in adulthood [42].

We assessed and compared the associations between an obesogenic DP at age 13, with a novel composite metabolomics score [31] vs. a conventional cardiometabolic risk (CMR) score assessed in 15-, 17-, and 24-years-old participants from the Avon Longitudinal Study of Parents and Children (ALSPAC)[46].

## Methodology

### Study population

**Fig. 1:**
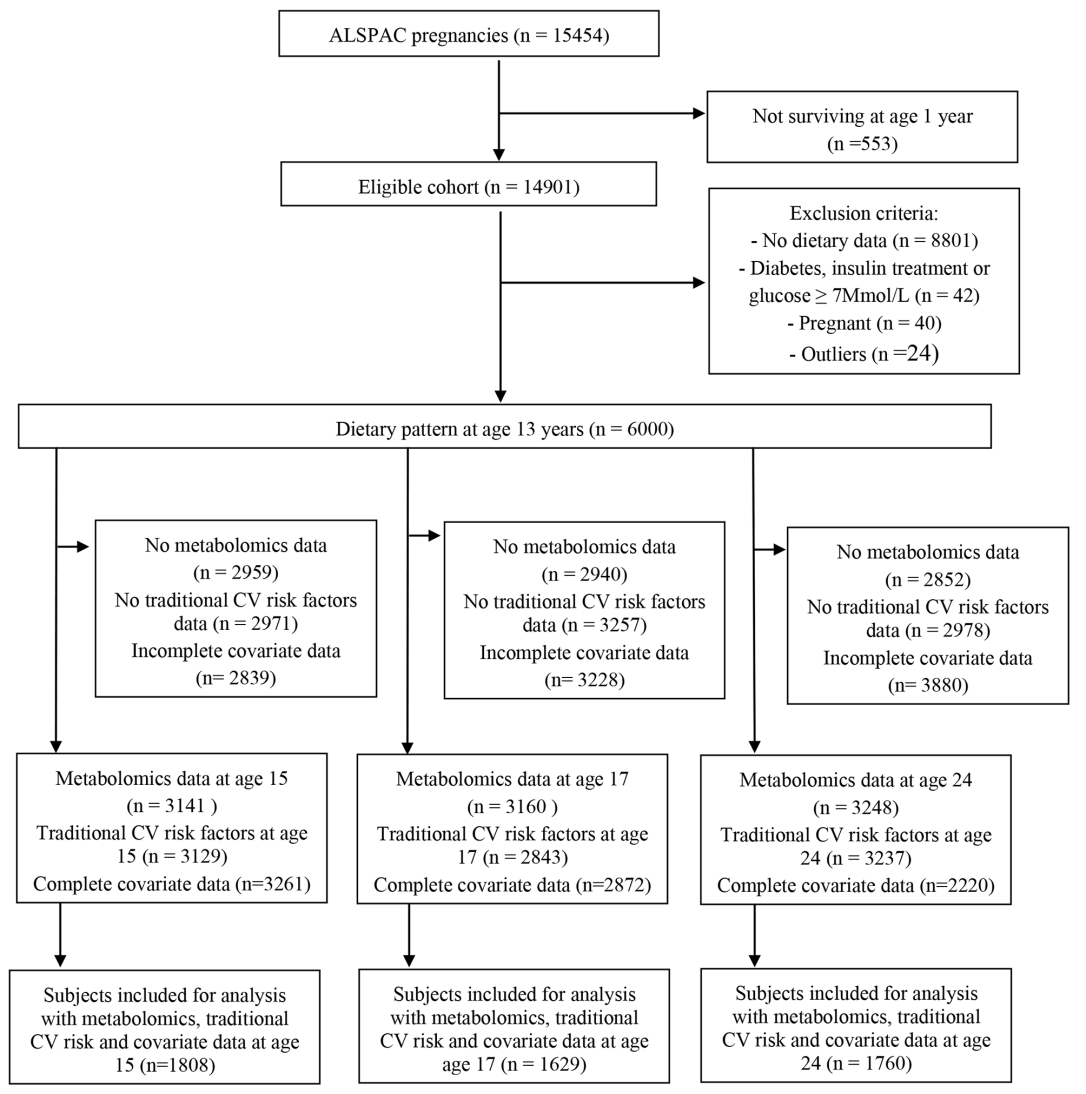
Flow chart of the study participants and reasons for exclusion from analyses

The data included in this study was obtained from participants recruited as part of the ALSPAC. The study enrolled pregnant women resident in Bristol in the South West of England with an expected delivery between 1st April 1991 and 31st December 1992, and included 14,541 eligible pregnant women from the South-West of England, resulting in 13,988 children alive at 1 year [44, 45, 46]. Two subsequent recruitment phases in 1999 and in 2012 provided a final sample of 15,454 pregnancies and 14,901 eligible children alive at 1 year [45] (Fig. 1). During periodic follow-ups, extensive data has been collected from the parents and their children, primarily using questionnaires, medical records and face to face visits. The study comprises a wide range of phenotypic, environmental, biological and epigenetic measures to investigate its effect on health. Study data were collected and managed using REDCap electronic data capture tools hosted at the University of Bristol [47]. REDCap (Research Electronic Data Capture) is a secure, web-based software platform designed to support data capture for research studies. The study website contains details of all the data that is available through a fully searchable data dictionary and variable search tool [48].

Ethics approval for the study was obtained from the ALSPAC Ethics and Law Committee and the Local Research Ethics Committee [49] and consent for biological samples has been collected in accordance with the Human Tissue Act (2004).

### Dietary quality assessment

Participants completed a three day food diary over two non-consecutive weekdays and one weekend day at the age of 13 years. Parental input on the diary was allowed and diaries were checked by a nutritionist in a clinic visit. The food diaries were coded and linked to food composition tables using DIDO, a programme developed at MRC Human Nutrition Research [50]. Nutrient intakes were calculated using McCance and Widdowson’s British food composition Table [51]. Intakes of individual foods were combined into predefined Cambridge Food Groups used in previous analyses of ALSPAC dietary data [52].

Ambrosini et al., [21] using RRR, identified a DP that explains the maximum variation in a set of response variables hypothesized to be on the pathway between food intake and obesity in children and adolescents. The RRR model included intakes of 46 food groups (g/d) as predictor variables and the following 4 response variables: energy density, % energy from fat and sugar, and dietary fibre density. Dietary energy density (kJ/g) was calculated as total food energy (kJ) divided by total food weight (g), excluding beverages [53]. Dietary fibre density (g/MJ) was calculated as total g fibre (nonstarch polysaccharide) divided by total energy intake (MJ). The % energy from fat was calculated as grams of total fat multiplied by 37 kJ then divided by total EI (kJ), and multiplied by 100. The % energy from sugar was calculated as grams of free sugar multiplied by 17 kJ then divided by total EI (kJ), and multiplied by 100.

The factor loading of the 46 food groups quantified the extent to which specific foods increased (if positive) or decreased (if negative) the energy density, fat, fibre or sugar content of the overall diet [21]. RRR produces a DP z-score as a linear, weighted combination of standardized food group intakes by their factor loadings. Therefore, each respondent received a z-score quantifying the degree to which their reported DP at 13 years was obesogenic with a higher DP z-score indicating a more obesogenic DP (lower in fibre and higher in fat, sugar and energy density) [21].

### Blood samples

Fasting blood samples were collected during clinic visits at ages 15, 17 and 24 years. Plasma and serum samples from each clinic were processed by the Bristol Bioresource Laboratories where they were centrifuged at 3500 rpm, 4–5 °C for 10 min. Plasma was subsequently aliquoted and stored temporarily at -20°C before long term storage at -70/80°C. Samples remained frozen for three to nine months, until they were plated out into 96 well plates for biomarker analysis using standard procedures, with no previous freeze-thaw cycles [54, 55].

### Metabolomics score

The metabolomic biomarkers used in this analysis were quantified in plasma using nuclear magnetic resonance from the Nightingale Health platform, which is a Finnish metabolomics programme specialized in health technology that has been used in numerous published epidemiological studies [56].

Using the Nightingale metabolomics platform, Deelen et al., [31] selected 14 metabolites from the 226 that were measured based on previous studies using metabolomics [57, 58]. The 14 metabolites were:

- total lipids in extremely large very low-density lipoprotein particles (VLDL) (diameter > 75.0 nm).
- total lipids in small HDL (diameter < 8.7 nm).
- VLDL diameter.
- ratio of polyunsaturated fatty acids to total fatty acids.
- glucose.
- lactate.
- histidine.
- isoleucine.
- leucine.
- valine.
- phenylalanine.
- acetoacetate.
- albumin.
- glycoprotein acetyls.

Each metabolite was standardized by creating z-scores and multiplied by the logarithm of the hazard ratio, which is based on the weight that each metabolite had on the risk of all-cause mortality in the study by Deelen et al.,(2019) (Additional file 1, Table S1). Subsequently, the score for each of the 14 metabolites was summed to obtain an overall continuous metabolomics score where a higher score indicates an unhealthier CMR profile [31].

### Traditional cardiometabolic risk score

The CMR score was based on a recent study from the ALSPAC cohort [43]. This score included six cardiometabolic markers: fat mass index (FMI), HDL cholesterol (HDL-c), LDL cholesterol (LDL-c), triacylglycerol, mean arterial pressure (MAP) and homeostatic model assessment of insulin resistance (HOMA-IR) which were collected during clinic visits when participants were 15, 17 and 24 years of age.

FMI was calculated by dividing fat mass (kg) measured with DXA by height (m^2^). Blood pressure (BP) was recorded in the right arm in seated position using an Omron M6 upper arm monitor and the following formula was used to calculate the MAP: (systolic BP + (2* diastolic BP))/3 [59]. HOMA-IR was calculated from fasting plasma glucose and insulin ((fasting plasma glucose (mg.dL-1) x fasting plasma insulin (mU.L-1))/405) [60]. Insulin was measured by an ultrasensitive ELISA (Mercodia, Uppsala, Sweden) whereas glucose values were converted from mmol/L to mg/dL and obtained by automated enzymatic (hexokinase) method [61]. Plasma lipids (LDL-c, triglycerides, and HDL-c) were performed by modification of the standard Lipid Research Clinics Protocol using enzymatic reagents for lipid determination [62].

To calculate the CMR score, sex-specific z-scores were calculated for each CMR marker in order to standardize the units for different risk factors. HDL-c was then multiplied by -1, to align the direction of values for increased risk with the other components. The z-scores from the six CMR components were summed to give the final CMR score for each participant at 15, 17 and 24 years. A higher CMR score represented a worse overall CMR profile.

### Covariates

Several covariates were included as potential confounders in our analyses, based on previous studies assessing cardiometabolic traits in children from ALSPAC [63, 64]. The covariates included were age, sex, dietary misreporting, highest household social class, maternal highest educational attainment, BMI and moderate-to-vigorous physical activity level (MVPA) at age 13.

Parental data were collected by self-completion postal questionnaires during pregnancy. Household social class was obtained from the highest of mother and partner social class, and was based on the occupational category and categorized into a dichotomous variable (higher and lower), as reported elsewhere [65]: I, II and III non-manual categories for the higher social class (professionals, managerial and technical occupations and non-manual skilled categories) and III-manual, IV and V categories for the lower social class (manual skilled, partly skilled and unskilled occupations). Maternal Educational level was categorized into (1) Certificate of Secondary Education, (2) vocational training, (3) O-level/General Certificate of Secondary Education (compulsory exams taken at age 16), (4) A-levels (optional exams taken at age 18) or degree or higher.

Dietary misreporting was based on the ratio of self-reported energy intake to estimated energy requirement (EI:EER) at age 13. Individuals were classified as plausible reporters (EI:EER within the 95% CI for EI:EER), over-reporters (EI:EER > 95% CI for EI:EER) or under-reporters (EI:EER < 95% CI EI:EER) [66]. Additionally, children who attended the clinic visits at age 13 were asked to wear an Actigraph AM7164 2.2 accelerometer (Actigraph LLC, Fort Walton Beach, FL, USA) on their right hip for 7 days. Participants were subsequently divided as physically active (> 60 min of moderate-to-vigorous PA/day) or inactive (< 60 min of moderate-to-vigorous PA/day). Finally, child height (m) and weight (kg) were measured in clinics and BMI was computed by dividing weight (kg) by height (m^2^). Obesity was defined using BMI cut-off points at age 13 based on the International Obesity Task Force values for children [67].

### Statistical analysis

Prior to the analysis, participants diagnosed with diabetes, undergoing insulin treatment, or with a fasting blood glucose concentration of ≥ 7Mmol/L (n = 42) were excluded due to issues of using HOMA-IR to assess insulin sensitivity in diabetic subjects [68]. In addition, participants (n = 24) with implausible/extreme outlier data (defined as more than 4 SDs from the mean) for the CMR score markers were excluded. Participants who were pregnant during the follow-up period (n = 40), and without dietary data at age 13 years (n = 8801) were also excluded from the analysis.

The characteristics of the participants (measured at age 13 or earlier) included in the analysis at age 15, 17 and 24 years are described using n (%). Continuous variables are described using means and 95% CI after confirming normality. Simple associations were estimated using univariable regression analysis of the continuous exposure (DP z-score) and outcome variables (CMR z-score, metabolomics z-score) with groups defined by covariates in samples included in analyses at age 15, 17 and 24. To assess missing data bias we compared participants included in the analysis at age 15 with those excluded. We tested the differences in groups defined by covariates using chi-square test and mean exposure and outcome z-scores using independent t-test. Missing data results were very similar at age 15, 17 and 24, so only data on age 15 is reported.

The adjusted association between DP score at age 13 years and the metabolomics score at age 15, 17, and 24 was assessed using multivariable linear regression models. The DP z-score was included in separate models both as a categorical independent variable, divided by tertiles (first tertile as reference), and as a continuous variable (per unit increase in the z-score). The same procedure was carried out to assess the association between DP score and the traditional CMR score.

Three different models were reported for the multivariable linear regression analyses: a crude model with just the exposure and outcome variables, a minimally adjusted model adjusting for age, sex and dietary misreporting, and a fully adjusted model additionally adjusting for highest household social class, highest maternal educational attainment, BMI and MVPA level at age 13. However, BMI was excluded from the traditional CMR score models because FMI was already one of the risk factors included in the score. Finally, we compared the association between DP z-score with metabolomics z-score and with the traditional CMR z-score using a Z-test. All the analyses were conducted using SPSS Statistics v27.

## Results

Our analysis included 1808 participants at 15 years old (mean age 15.5, SD 0.3), 1629 participants at 17 years old (mean age 17.8, SD 0.4), and 1760 at 24 years old (mean age 24.5, SD 0.8) (Fig. 1 details all prior exclusions).

The characteristics of the study population and associations of the metabolomics and traditional CMR score with covariates are presented in Tables 1 and 2, respectively. There were 53.7% female participants, 60.2% with I, II or III non-manual social class, 50.5% A-level/degree or higher educated mothers, 65.9% physically inactive, 22% with obesity or overweight, and 37.4% plausible reporters at baseline. Compared to the excluded sample, participants included were more likely to be females, have a higher household social class, higher maternal educational level, less likely to be overweight or obese, and had lower mean DP, metabolomics, and CMR z-scores (Additional file 1, Table S2).

**Table 1:** Associations of metabolomics z-score with baseline characteristics of participants included in analysis

	Participants at age 15 (n = 1808)	Metabolomics z-score at age 15		Participants at age 17 (n = 1629)	Metabolomics z-score at age 17		Participants at age 24 (n = 1760)	Metabolomics z-score at age 24	
	n (%)	Mean (95% CI)	P trend	n (%)	Mean (95% CI)	P trend	n (%)	Mean (95% CI)	P trend
Sex									
Male	838 (46.3)	Ref		781 (47.9)	Ref		732 (41.6)	Ref	
Female	970 (53.7)	-0.08 (-0.15, 0.00)	0.071	848 (52.1)	-0.01 (-0.19, 0.00)	0.053	1028 (58.4)	0.1 (0.00, 0.20)	0.034
Social class ^a^									
I, II, III non-manual	1089 (60.2)	Ref		1018 (62.5)	Ref		1120 (63.6)	Ref	
III manual, IV, V	719 (39.8)	0.07 (-0.02, 0.16)	0.140	611 (37.5)	0.40 (-0.06, 0.14)	0.432	640 (36.4)	0.09 (0.00, 0.19)	0.076
Maternal educational level ^b^									
A-level or degree	914 (50.5)	Ref		860 (52.8)	Ref		935 (53.1)	Ref	
O-level	603 (33.4)	0.12 (0.15, 0.22)		518 (31.8)	0.14 (0.03, 0.25)		583 (33.1)	0.18 (0.08, 0.28)	
Vocational	128 (7.1)	-0.03 (-0.22, 0.15)		113 (6.9)	-0.08 (-0.28, 0.11)		101 (5.7)	0.02 (-0.18, 0.23)	
CSE or none	163 (9.0)	0.02 (-0.14, 0.19)	0.125	138 (8.5)	0.10 (-0.14, 0.19)	0.035	141 (8.0)	0.10 (-0.09, 0.27)	< 0.001
Physical activity level age 13^c^									
Inactive (< 60 MVPA/day)	1191 (65.9)	Ref		1077 (66.1)	Ref		1218 (69.2)	Ref	
Active (≥ 60 MVPA/day)	617 (34.1)	-0.02 (-0.12, 0.07)	0.610	552 (33.9)	0.00 (-0.10, 0.10)	0.961	542 (30.8)	-0.02 (-0.12, 0.08)	0.658
BMI ^d^									
Obese	76 (4.2)	0.42 (0.19, 0.65)		63 (3.9)	0.44 (0.18, 0.69)		72 (4.1)	0.58 (0.34, 0.82)	
Overweight	322 (17.8)	0.08 (-0.04, 0.20)		269 (16.6)	0.05 (-0.08, 0.18)		301 (17.1)	0.19 (0.06, 0.31)	
Normal Underweight	1322 (73.2) 85 (4.7)	Ref 0.12 (-0.09, 0.34)	0.003	1212 (74.6) 81 (5.0)	Ref -0.14 (-0.37, 0.08)	0.003	1287 (73.1) 101 (5.7)	Ref -0.07 (-0.27, 0.13)	< 0.001
Dietary misreporting ^e^									
Over-reporting	19 (1.1)	0.43 (-0.03, 0.88)		16 (1.0)	-0.16 (-0.65, 0.34)		13 (0.7)	-0.01 (-0.55, 0.53)	
Plausible reporting	677 (37.4)	Ref		640 (39.4)	Ref		697 (39.6)	Ref	
Under-reporting	1112 (61.5)	0.03 (-0.06, 0.12)	0.174	973 (59.6)	0.08 (-0.02, 0.18)	0.23	1050 (59.7)	0.12 (0.02, 0,22)	0.051

^a^ I, II, III non-manual social class categories correspond to the highest one, and III manual, IV and V to the lowest. ^b^ A-level or degree correspond to the highest maternal educational level, and CSE or none to the lowest .^c^ Sufficient physical activity data was only available at age 13. ^d^ BMI sex-specific cut off points were obtained at age 13 from the International Obesity Task Force.^e^ Dietary misreporting was based on the ratio of energy intake to estimated energy requirement (EER) at age 13. Abbreviations = BMI: Body mass index. MVPA : Moderate-to-vigorous physical activity

**Table 2:** Traditional CMR z-score and baseline characteristics of participants included in analysis

	Participants at age 15^a^ (n = 1808)	CMR z-score at age 15		Participants at age 17^a^ (n = 1629)	CMR z-score at age 17		Participants at age 24^a^ (n = 1760)	CMR z-score at age 24	
	n (%)	Mean (95% CI)	P trend	n (%)	Mean (95% CI)	P trend	n (%)	Mean (95% CI)	P trend
Sex									
Male Female	970 (53.7) 838 (46.3)	Ref 0.34 (0.25, 0.43)	< 0.001	781 (47.9) 848 (52.1)	Ref 0.23 (0.14, 0.32)	< 0.001	732 (41.6) 1028 (58.4)	Ref -0.19 (-0.29,-0.10)	< 0.001
Social class^a^									
I, II, III non-manual III manual, IV, V	1089 (60.2) 719 (39.8)	Ref 0.03 (-0.06, 0.13)	0.460	1018 (62.5) 611 (37.5)	Ref 0.13 (0.04, 0.23)	< 0.001	1120 (63.6) 640 (36.4)	Ref 0.15 (0.05, 0.24)	< 0.001
Maternal educational level^b^									
A-level or degree O-level Vocational CSE or none	914 (50.5) 603 (33.4) 128 (7.1) 163 (9.0)	Ref 0.12 (0.02, 0.22) 0.01 (-0.18, 0.19) 0.08 (-0.08, 0.25)	0.135	860 (52.8) 518 (31.8) 113 (6.9) 138 (8.5)	Ref 0.17 (0.06, 0.27) 0.11 (-0.07, 0.30) 0.27 (0.09, 0.44)	< 0.001	935 (53.1) 583 (33.1) 101 (5.7) 141 (8.0)	Ref 0.25 (0.15, 0.35) 0.30 (0.10, 0.50) 0.21 (0.04, 0.39)	< 0.001
Physical activity level age 13^c^									
Inactive (< 60 MVPA/day) Active (≥ 60 MVPA/day)	1191 (65.9) 617 (34.1)	Ref -0.31 (-0.41,-0.22)	< 0.001	1077 (66.1) 552 (33.9)	Ref -0.19 (-0.28,-0.09)	< 0.001	1218 (69.2) 542 (30.8)	Ref -0.07 (-0.17, 0.03)	0.198
Dietary misreporting^d^									
Over-reporting Plausible reporting Under-reporting	19 (1.1) 677 (37.4) 1112 (61.5)	-0.50 (-0.94,-0.05) Ref 0.28 (0.18, 0.37)	< 0.001	16 (1.0) 640 (39.4) 973 (59.6)	-0.39 (-0.86, 0.08) Ref 0.30 (0.21, 0.40)	< 0.001	13 (0.7) 697 (39.6) 1050 (59.7)	-0.10 (0.65, 0.45) Ref 0.24 (0.15, 0.34)	< 0.001

^a^I, II, III non-manual social class categories correspond to the highest one, and III manual, IV and V to the lowest.^b^ A-level or degree correspond to the highest maternal educational level, and CSE or none to the lowest .^c^ Sufficient physical activity data was only available at age 13. ^d^ Dietary misreporting was based on the ratio of energy intake to estimated energy requirement (EER) at age 13. BMI was excluded from this analysis because FMI is included in the conventional CMR score. Abbreviations = BMI: Body mass index; CMR: Cardiometabolic risk; MVPA: Moderate-to-vigorous physical activity

We found an association between a higher metabolomics z-score and being overweight or obese across all ages, a lower maternal education at ages 17 and 24, and being female at age 24 years (Table 1). In contrast, having a higher traditional CMR score was associated with a lower household social class,  lower maternal education, greater under-reporting of energy intake across all ages, being female at 13 and 15 years old, being male at 24 years, and inactivity at age 15 and 17, but not 24 years (Table 2). A more obesogenic DP score was associated with a lower household social class, lower maternal educational attainment, being male, and over-reporting energy intake (Additional file 1, Table S3). There was no cross-sectional evidence of an association between physical activity or weight status at age 13 years.

### Association of the obesogenic DP z-score with metabolomics score and traditional CMR z-score

After adjusting for confounders, being in the medium or the highest tertile of the DP z-score, compared to the lowest tertile, was associated at age 15 with an increase in the metabolomics z-score (β = 0.20; 95% CI 0.09–0.32 and β = 0.15; 95% CI 0.04–0.26, respectively) (Table 3) and with the traditional CMR z-score (β = 0.15; 95% CI 0.05–0.24 for highest versus lowest DP z-score and β = 0.11; 95% CI 0.01–0.20 for medium versus lowest DP z-score) (Table 4). Additionally, there was evidence of a positive association between the continuous DP z-score at age 13 and the metabolomics z-score at age 15 (β = 0.06; 95% CI 0.03–0.10), but no evidence was found for the traditional CMR score.

**Table 3:** Association between dietary pattern z-score and metabolomics z-score at age

		Metabolomics z-score											
			Crude						Adjusted (model 1)^a^			Adjusted (model 2)^b^	
	N		Beta (95% CI)			P trend			Beta (95% CI)	P trend		Beta (95% CI)	P trend
Age 15													
Low DP z-score^c^	608		Reference						Reference			Reference	
Medium DP z-score	596		0.15 (0.04, 0.26)						0.15 (0.04, 0.26)			0.15 (0.04, 0.26)	
High DP z-score	604		0.22 (0.11, 0.34)				< 0.001		0.22 (0.10, 0.33)	< 0.001		0.20 (0.09, 0.32)	< 0.001
Continuous DP z-score	1808		0.06 (0.03, 0.10)				< 0.001		0.06 (0.03, 0.10)	< 0.001		0.06 (0.03, 0.10)	< 0.001
Age 17													
Low DP z-score	548		Reference						Reference			Reference	
Medium DP z-score	537		0.03 (-0.09, 0.15)						0.03 (-0.08, 0.15)			0.04 (-0.08, 0.16)	
High DP z-score	544		0.20 (0.10, 0.31)				< 0.001		0.24 (0.12, 0.36)	< 0.001		0.22 (0.10, 0.34)	< 0.001
Continuous DP z-score	1629		0.05 (0.02, 0.09)				< 0.001		0.06 (0.02, 0.09)	< 0.001		0.05 (0.02, 0.09)	< 0.001
Age 24													
Low DP z-score	590		Reference						Reference			Reference	
Medium DP z-score	579		0.11 (-0.01, 0.22)						0.10 (-0.01, 0.22)			0.08 (-0.03, 0.19)	
High DP z-score	591		0.11 (-0.01, 0.23)				0.027		0.15 (0.03, 0.27)	0.005		0.11 (-0.01, 0.22)	0.027
Continuous DP z-score	1760		0.02 (-0.01, 0.05)				0.197		0.03 (0.00, 0.06)	0.048		-0.01 (-0.05, 0.03)	0.194

^a^ Adjusted for sex, age (at outcome, when the score was measured) and dietary misreporting. ^b^Adjusted for sex, age, dietary misreporting, maternal and paternal social class, maternal educational level, physical activity (PA) level (average minutes of moderate-to-vigorous PA per day) at age 13, and body mass index at each correspondent age. ^c^ All categories were obtained from the dietary pattern z-score tertiles at each age: Low = First tertile. Medium = Second tertile. High = third tertile .Abbreviations = DP: Dietary pattern

**Table 4:** Association between dietary pattern score with the traditional CMR z-score

		CMR z-score										
			Crude					Adjusted (model 1)^a^			Adjusted (model 2)^b^	
	N		Beta (95% CI)			P trend		Beta (95% CI)	P trend		Beta (95% CI)	P trend
Age 15												
Low DP z-score^c^	608		Reference					Reference			Reference	
Medium DP z-score	596		0.11 (0.01, 0.20)					0.13 (0.02, 0.24)			0.11 (0.01, 0.20)	
High DP z-score	604		-0.02 (-0.12, 0.07)			0.425		0.14 (0.03, 0.25)	0.012		0.15 (0.05, 0.24)	0.020
Continuous DP z-score	1808		0.00 (-0.03, 0.03)			0.847		0.03 (0.00, 0.06)	0.088		0.02 (-0.01, 0.05)	0.132
Age 17												
Low DP z-score	548		Reference					Reference			Reference	
Medium DP z-score	537		-0.01 (-0.12, 0.11)					0.01 (-0.10, 0.12)			0.02 (-0.06, 0.11)	
High DP z-score	544		0.07 (-0.05, 0.18)			0.239		0.17 (0.05, 0.28)	0.004		0.07 (-0.03, 0.16)	0.137
Continuous DP z-score	1629		0.00 (-0.03, 0.03)			0.737		0.04 (0.00, 0.07)	0.025		0.01 (-0.01, 0.04)	0.289
Age 24												
Low DP z-score	590		Reference					Reference			Reference	
Medium DP z-score	579		0.03 (-0.08, 0.14)					0.02 (-0.09, 0.13)			0.01 (-0.07, 0.09)	
High DP z-score	591		0.08 (-0.03, 0.19)			0.161		0.11 (0.00, 0.23)	0.053		0.03 (-0.06, 0.11)	0.435
Continuous DP z-score	1760		0.02 (-0.01, 0.05)			0.154		0.03 (0.00, 0.06)	0.034		0.01 (-0.01, 0.03)	0.496

^a^Adjusted for sex, age (at outcome, when the score was measured) and dietary misreporting. ^b^Adjusted for sex, age, dietary misreporting, maternal and paternal social class, maternal educational level, physical activity (PA) level (average minutes of moderate-to-vigorous PA per day) at age 13. ^c^ All categories were obtained from the dietary pattern z-score tertiles at each age: Low = First tertile. Medium = Second tertile. High = third tertile. Abbreviations = CMR: Cardiometabolic risk. DP: Dietary pattern

**Fig. 2:**
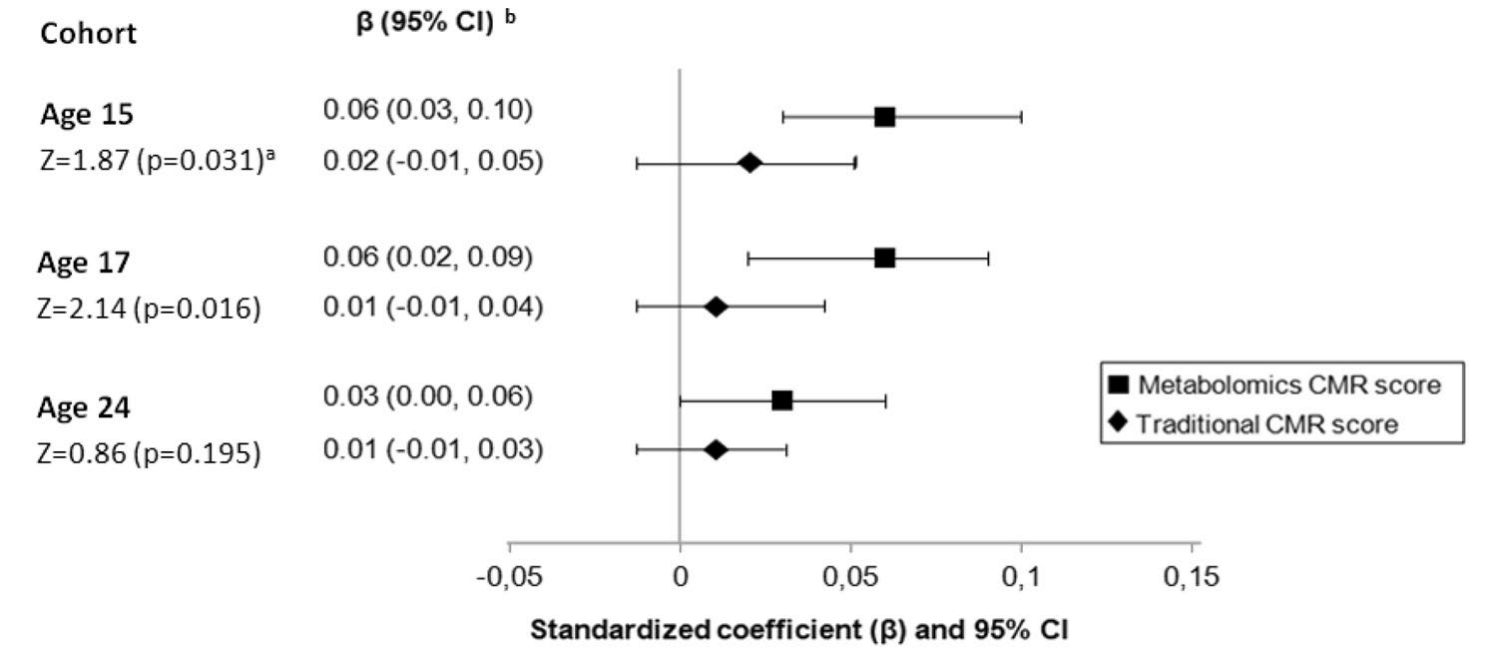
Z-test between DP z-score and the metabolomics and traditional CMR z-scores at age 15, 17 and 24. ^a^ Z-test was used to compare the association of DP z-score with metabolomics z-score and with the traditional CMR z-score. The formula is z = (x – µ) / (σ√n). x = sample mean, µ = population mean, σ = population standard deviation, n = sample size. ^b^Estimates were obtained from regression models between DP score at age 13 years and the metabolomics and conventional CMR score at age 15, 17, and 24 and adjusted for sex, age, dietary misreporting, maternal and paternal social class, maternal educational level, physical activity level (average minutes of moderate-to-vigorous PA per day) at age 13 for both scores, plus body mass index at each correspondent age for the metabolomics score. Abbreviations: CMR = Cardiometabolic risk

Being in the highest tertile of DP z-score, compared to the lowest, was also associated with an increase in metabolomics z-score at age 17 (β = 0.22; 95% CI 0.10–0.34) in the fully adjusted model (Table 3). However, no association was found between DP z-score and traditional CMR z-score at this age (Table 4). There was no association between DP z-score and either cardiometabolic z-score at age 24. Finally, there was evidence that the association between the DP z-score and metabolomics z-score was stronger than between the DP z-score and the traditional CMR z-score at ages 15 years (p = 0.031) and 17 years (p = 0.016), but not 24 years (Fig. 2).

## Discussion

An obesogenic DP score at age 13 years, characterized by high energy-density, high total fat and free sugars, and low fibre density, was associated with a worse cardiometabolic profile at 15 and 17 years old when assessed using a novel metabolomics score consisting of 14 plasma metabolites. The obesogenic DP score at 13 years was associated with a worse composite CMR score based on six conventional risk markers (FMI, HDL-c, LDL-c, TAG, MAP, and HOMA-IR) at 15 years, but not at 17 or 24 years. Our results suggest a stronger association between the DP score and the metabolomics score, compared to the conventional CMR score. To our knowledge, this is the first prospective study to find that an obesogenic DP in adolescence is associated with worse cardiometabolic profile, using a multi-marker CMR metabolomic score in a large population of adolescents with three different outcome time points.

A longitudinal study from the Western Australian Pregnancy Cohort identified a very similar ‘low fibre, high-energy density, high fat and sugar intake’ DP that was associated with conventional CMR factors (glucose, waist circumference, BMI, insulin, HDL-c, LDL-c, triglycerides) in 14 and 17-years-old adolescents [10]. As opposed to our study, this study did not include a metabolomics CMR score and did not track CMR risk from adolescence to young adulthood. In addition, they used some different risk factors for computing the traditional CMR score, and it was conducted in a different population group, thus their findings may not be completely comparable with our study. In contrast to their findings, we only found evidence of an association between the DP and the traditional CMR score for the highest versus lowest tertile at age 15, and not at age 17 (or age 24), and not when the DP was modelled as a continuous score, suggesting that there was weak evidence of an association for the conventional CMR score.

Our findings showing a stronger association between the DP score and the metabolomics score is consistent with previous longitudinal studies on incident type-2 diabetes [69] and CVD [70, 71] which identified metabolite patterns using metabolomics with higher predictive power than conventional risk factors. Metabolomics might improve the identification of subtle metabolic variation from early-stage pathophysiological processes [72, 73], which could explain why stronger evidence was found for the metabolite score when compared to traditional risk factors which are typically still within a healthy range during adolescence. However, as opposed to our analysis, these studies were conducted on adults and did not evaluate the relationship between DPs and CMR.

We did not find evidence of a relationship between the obesogenic DP at age 13 years and the metabolomics or the conventional CMR score at age 24 years. These findings are in line with The Northern Ireland Young Hearts Study which included participants at age 12–15 years and followed-up at 20–25 years and did not observe any longitudinal associations between a Mediterranean DP score and individual CMR factors [74]. However, a recent prospective analysis from ALSPAC found that a higher Mediterranean-style diet score at age 13 years was associated with a better CMR profile at age 24 [43]. In addition, a cross-sectional analysis in young adults within the Raine cohort study (mean age 24.3 years) found that a similar ‘energy-dense, high fat and sugar, low fibre’ DP was associated with a higher BMI [75], which is known to be a CMR factor [76]. Differences between the DP scores, study designs, and food intakes within each cohort may explain these discrepancies. The lack of association at age 24 years found in our study could be explained because dietary data was measured at age 13 years, and the period from adolescence to young adulthood is a period of transition in eating behaviour [77, 78]. Therefore, diet measurement at age 13 years might no longer reflect how the young adults are eating and could explain why no evidence was found for an association between the DP at age 13 and the metabolomics or traditional CMR scores at age 24.

The DP score used in this study has its own limitations. It was calculated from diet diary data, relying on the participant’s response which has known measurement error, including self-reporting bias [79, 80]. However, diet diaries are less prone to misreporting than food frequency questionnaires [81], and we estimated the plausibility of dietary reporting and adjusted for this in all multivariable regression analyses [66].

A further limitation common to large prospective cohort studies was follow-up bias, because participants included in the current analysis were more likely to be female, have a higher household social class and maternal educational level, and were less likely to be overweight and had lower obesogenic DP scores and better CMR profiles, compared to those with incomplete dietary and covariate data. In addition, a previous study in ALSPAC found that dietary patterns during childhood are associated with several socioeconomic factors, meaning that children with less healthy diets were probably underrepresented in our final study sample [82]. This may affect the transferability of the study findings to the overall population, although we adjusted for confounders which were previously found to influence the association between DP and CMR factors among participants from ALSPAC [21, 64, 83]. Nonetheless, we cannot rule out residual confounding due to inherent bias of observational design studies.

This study has several strengths. Due to its prospective design, we were able to investigate the effect of an obesogenic DP on CMR with 3 repeated measures of outcomes. CMR was assessed at 15, 17 and 24 years which allowed us to evaluate the extent to which this DP at age 13 is associated with CMR throughout adolescence to young adulthood in a relatively large sample. Measures of cardiovascular and metabolic risk, including obesity, dyslipidaemia, elevated glucose and blood pressure, cluster together in children and adolescents [84–87]. Therefore, the use of CMR scores provides a more useful summary of overall cardiometabolic health than single risk factors for predicting and preventing CMR. CMR scores are also helpful when analyzing cardiometabolic health in children as they accumulate subtle variation in a range of risk factors that could be too little to show risk on their own in pediatric populations [84, 88]. The potential application of metabolomics in identifying CMR is well established, as it provides a comprehensive insight into pathophysiological mechanisms of diseases [30, 89]. However, to our knowledge, this study is the first to assess the effect of a DP on both metabolomics and conventional CMR scores. Using DPs, rather than isolated nutrients or foods, may better inform about diet-disease associations as they consider the possible interactions between nutrients and foods [11], and it has been suggested that the use of nutrient densities (e.g. energy density, fibre density and % energy from fat) can reduce the error linked to this dietary assessment method [90]. Finally, using a RRR-derived DP may be better at identifying a DP that explains disease-specific variation in dietary habits, compared to using completely *a priori* dietary assessment methods [91].

## Conclusions

Our findings suggest that having an obesogenic DP at age 13, characterized by high energy-density, high % of energy from total fat and free sugars, and low fibre density, is associated with higher CMR at both 15 and 17 years of age. However, no evidence of an association for any of the CMR scores was observed at age 24. We found stronger evidence of an association between the DP and CMR using a multimarker metabolomic score, compared to a traditional CMR score. These findings suggest the importance of avoiding an obesogenic DP during early puberty for future cardiometabolic health during later adolescence, and the utility of metabolomics for assessing diet and CMR relationships in epidemiological research in adolescents. Nonetheless, further research in trials is needed to establish a causal relationship.

### Electronic supplementary material

Below is the link to the electronic supplementary material.

**Additional file 1: Table S1**: Weight that each metabolite had on the risk of all-cause mortality (Deelen et al, 2019). **Table S2**: Description of the cohort included in analysis at age 15 (n=1808) and comparison with the participants not included from the ALSPAC cohort. Table S3: Description of age 13 year DP score by covariate groups among samples with outcome variable data at age 15, 17 and 24 years

## Data Availability

The data that support the findings of this study are available from the Avon Longitudinal Study of Parents and Children but restrictions apply to the availability of these data, which were used under license for the current study, and so are not publicly available. Data are however available from the authors upon reasonable request and with permission of ALSPAC. Researchers can apply to ALSPAC for use of the data. The study website (http://www.bristol.ac.uk/alspac/researchers/our-data/) contains details of all the data that are available through a fully searchable data dictionary and variable search tool.

## Abbreviations

CMR: Cardiometabolic risk
DP: Dietary pattern
RRR: Reduced rank regression
BMI: body mass index
ALSPAC: Avon Longitudinal Study of Parents and Children
HDL-c: High-density lipoprotein cholesterol
LDL-c: Low-density lipoprotein cholesterol
HOMA-IR: Homeostatic model assessment of insulin resistane
FMI: Fat mass index
MAP: Mean arterial pressure
CVD: Cardiovascular diseases
MVPA: Moderate-to-vigorous physical activity
PA: Physical activity
