# Supplementary material for: Prospective association between an obesogenic dietary pattern in early adolescence and metabolomics derived and traditional cardiometabolic risk scores in adolescents and young adults from the ALSPAC cohort
Source: Nutr Metab (Lond). 2023 Sep 15;20:41. doi: 10.1186/s12986-023-00754-z (PMC10504726; doi:10.1186/s12986-023-00754-z)
Supplement: Supplementary file 1 — Additional file 1: Table S1: Weight that each metabolite had on the risk of all-cause mortality (Deelen et al, 2019). Table S2: Description of the cohort included in analysis at age 15 (n=1808) and comparison with the participants not included from the ALSPAC cohort. Table S3: Description of age 13 year DP score by covariate groups among samples with outcome variable data at age 15, 17 and 24 years [file 12986_2023_754_MOESM1_ESM.docx]

# Supplementary material

Table S1. Weight that each metabolite had on the risk of all-cause mortality (Deelen et al, 2019)

| **Biomarker** | **Full name** | **HR** | **95% CI** | **P-value** |
| --- | --- | --- | --- | --- |
| XXL-VLDL-L | Total lipids in chylomicrons and extremely large VLDL | 0.80 | 0.75-0.85 | 1.53 x 10^-13^ |
| S-HDL-L | Total lipids in small HDL | 0.87 | 0.84-0.90 | 5.98x10^-19^ |
| VLDL-D | Mean diameter for VLDL particles | 0.85 | 0.80-0.90 | 8.51x10^-8^ |
| PUFA/FA | Ratio of polyunsaturated fatty acids to total fatty acids (%) | 0.78 | 0.75-0.80 | 1.06x10^-47^ |
| Glc | Glucose | 1.16 | 1.13-1.19 | 2.22x10^-29^ |
| Lac | Lactate | 1.06 | 1.03-1.10 | 6.24x10^-5^ |
| His | Histidine | 0.93 | 0.90-0.96 | 1.15x10^-5^ |
| Ile | Isoleucine | 1.23 | 1.14-1.32 | 2.14x10^-8^ |
| Leu | Leucine | 0.82 | 0.76-0.89 | 7.34x10^-7^ |
| Val | Valine | 0.87 | 0.82-0.92 | 1.04x10^-6^ |
| Phe | Phenylalanine | 1.13 | 1.09-1.17 | 2.39x10^-12^ |
| AcAce | Acetoacetate | 1.08 | 1.05-1.11 | 1.73x10^-8^ |
| Alb | Albumin | 0.89 | 0.87-0.92 | 9.96x10^-13^ |
| GlycA | Glycoprotein acetyls | 1.32 | 1.27-1.38 | 7.45x10^-41^ |

HR: Hazard ratio. CI: Confidence interval. VLDL: very low-density lipoprotein particle. HDL: high-density lipoprotein.

Table S2. Description of the cohort included in analysis at age 15

(n=1808) and comparison with the participants not included from the ALSPAC cohort

|  | **Participants not included at age 15** |  | **Included participants at age 15** | **p-value^a^** |
| --- | --- | --- | --- | --- |
|  | **n (%)** |  | **n (%)** |  |
| Sex |  |  |  |  |
| Female | 6578 (48.3) |  | 970 (53.7) |  |
| Male | 7041 (51.7) |  | 838 (46.3) | <0.001 |
| Household social class |  |  |  |  |
| I, II, III non-manual | 3953 (47.4) |  | 1089 (60.2) |  |
| III manual, IV, V | 4382 (52.6) |  | 719 (39.8) | <0.001 |
| Maternal education level |  |  |  |  |
| A-level or degree | 2845 (30.8) |  | 914 (50.5) |  |
| O-level | 3199 (34.6) |  | 603 (33.4) |  |
| Vocational | 988 (10.7) |  | 128 (7.1) |  |
| CSE or none | 2215 (24.0) |  | 163 (9.0) | <0.001 |
| BMI |  |  |  |  |
| Obese | 159 (5.5) |  | 76 (4.2) |  |
| Overweight | 581 (20.1) |  | 322 (17.8) |  |
| Normal | 2002 (69.1) |  | 1325 (73.2) |  |
| Underweight | 154 (5.3) |  | 85 (4.7) | 0.023 |
| Dietary misreporting |  |  |  |  |
| Over-reporting | 41 (1.5) |  | 19 (1.1) |  |
| Valid reporting | 987 (34.9) |  | 677 (37.4) |  |
| Under-reporting | 1793 (63.5) |  | 1112 (61.5) | 0.078 |
|  | **Mean (SD)** |  | **Mean (SD)** | **P-value^b^** |
| Dietary pattern z-score^c^ | -0.07 (1.54) |  | -0.20 (1.46) | <0.001 |
| Traditional CMR z-score^d^ | 0.04 (0.99) |  | -0.04 (0.99) | 0.052 |
| Metabolomics z-score^d^ | 0.07 (1.00) |  | -0.05 (0.99) | <0.001 |

^a^Chi-squared test used to test differences between excluded and included sample characteristics. ^b^Independent t tests test used to test differences between excluded and included exposures and outcomes. ^c^Standardized by the dietary pattern score at dietary data collection (age 13). ^d^Standardized by the relevant score at age 15. Abbreviations: Sd: Standard deviation. Abbreviations: BMI = Body mass index. SD = Standard deviation. CMR = Cardiometabolic risk.

Table S3. Description of age 13 year DP score by covariate groups among samples with outcome variable data at age 15, 17 and 24 years

|  | **Participants at age 15**  **(n=1808)** | **DP z-score at age 15** | | **Participants at age 17**  **(n=1629)** | **DP z-score at age 17** | | **Participants at age 24**  **(n=1760)** | **DP z-score at age 24** | |
| --- | --- | --- | --- | --- | --- | --- | --- | --- | --- |
|  | **n (%)** | **Mean (95% CI)** | **P trend** | **n (%)** | **Mean (95% CI)** | **P trend** | **n (%)** | **Mean (95% CI)** | **P trend** |
| Sex^a^  Male  Female | 838 (46.3)  970 (53.7) | Ref  -0.23 (-0.37, -0.10) | <0.001 | 781 (47.9)  848 (52.1) | Ref  -0.33 (-0.48, -0.19) | <0.001 | 732 (41.6)  1028 (58.4) | Ref  -0.34 (-0.49, -0.20) | <0.001 |
| Social class^b^  I, II, III non-manual  III manual, IV, V | 1089 (60.2) 719 (39.8) | Ref  0.31 (0.17, 0.45) | <0.001 | 1018 (62.5)  611 (37.5) | Ref  0.33 (0.18, 0.48) | <0.001 | 1120 (63.6)  640 (36.4) | Ref  0.25 (0.11, 0.40) | <0.001 |
| Maternal educational level^c^  A-level or degree  O-level  Vocational  CSE or none | 914 (50.5) 603 (33.4) 128 (7.1)  163 (9.0) | Ref  0.31 (0.16, 0.46)  0.48 (0.21, 0.75)  0.68 (0.44, 0.92) | <0.001 | 860 (52.8)  518 (31.8)  113 (6.9)  138 (8.5) | Ref  0.37 (0.21, 0.53)  0.51 (0.21, 0.80)  0.64 (0.37, 0.90) | <0.001 | 935 (53.1)  583 (33.1)  101 (5.7)  141 (8.0) | Ref  0.31 (0.16, 0.47)  0.54 (0.23, 0.84)  0.56 (0.30, 0.83) | <0.001 |
| Physical activity level |  | Ref  0.04 (-0.10, 0.18) |  | 1077 (66.1)  552 (33.9) | Ref  0.10 (-0.05, 0.26) | 0.183 | 1218 (69.2)  542 (30.8) | Ref  0.07 (-0.08, 0.23) | 0.340 |
| Inactive (≥60 MVPA/day)  Active (<60 MVPA/day) | 1191 (65.9)  617 (34.1) |  | 0.581 |  |  |  |  |  |  |
| BMI^d^  Obese  Overweight  Normal  Underweight | 76 (4.2)  322 (17.8)  1322 (73.2)  85 (4.7) | -0.02 (-0.36, 0.32)  -0.07 (-0.25, 0.10)  Ref  0.14 (-0.18, 0.46) |  | 63 (3.9)  269 (1.6)  1212 (74.6)  81 (5.0) | -0.03 (-0.41, 0.35)  -0.04 (-0.23, 0.16)  Ref  0.17 (-0.17, 0.51) |  | 72 (4.1)  301 (17.1)  1287 (73.1)  101 (5.7) | -0.23 (-0.59, 0.13)  -0.12 (-0.31, 0.06)  Ref  0.32 (0.01, 0.62) |  |
|  |  |  | 0.674 |  |  | 0.746 |  |  | 0.047 |
| Dietary misreporting^e^  Over-reporting  Plausible reporting  Under-reporting | 19 (1.1)  677 (37.4)  1112 (61.5) | 1.05 (0.40, 1.71)  Ref  -0.55 (-0.68, -0.41) | <0.001 | 16 (1.0)  640 (39.4)  973 (59.6) | 1.44 (0.71, 2.17)  Ref  -0.52 (-0.67, -0.37) | <0.001 | 13 (0.7)  697 (39.6)  1050 (59.7) | 1.68 (0.88, 2.45)  Ref  -0.57 (-0.71, -0.43) | <0.001 |

^a^ Data from all independent variables included in the table was collected at age 13. ^b^ I, II, III non-manual social class categories correspond to the highest, and III manual, IV and V to the lowest. ^c^ A-level or degree correspond to the highest maternal educational level, and CSE or none to the lowest . ^d^ BMI sex-specific cut off points were obtained from the International Obesity Task Force ^e^Dietary misreporting was based on the ratio of energy intake to estimated energy requirement (EER) at age 13. Abbreviations = BMI: Body mass index. DP: Dietary pattern. MVPA = Moderate-to-vigorous physical activity
